# Varicella-zoster virus proteome-wide T-cell screening demonstrates low prevalence of virus-specific CD8 T-cells in latently infected human trigeminal ganglia

**DOI:** 10.1186/s12974-023-02820-y

**Published:** 2023-06-12

**Authors:** Michiel van Gent, Werner J. D. Ouwendijk, Victoria L. Campbell, Kerry J. Laing, Georges M. G. M. Verjans, David M. Koelle

**Affiliations:** 1 HerpesLabNL, Department of Viroscience, Erasmus Medical Center, Dr. Molewaterplein 40, 3015 GD Rotterdam, The Netherlands; 2 Department of Medicine, University of Washington, Seattle, WA 98195 USA; 3 Department of Laboratory Medicine and Pathology, University of Washington, Seattle, WA 98195 USA; 4 Vaccine and Infectious Diseases Division, Fred Hutchinson Cancer Center, Seattle, WA 98109 USA; 5 Department of Global Health, University of Washington, Seattle, WA 98195 USA; 6 Department of Translational Research, Benaroya Research Institute, Seattle, WA 98101 USA

**Keywords:** Varicella-zoster virus, Herpes simplex virus, Human, Trigeminal ganglion, Latency, T-cells

## Abstract

**Background:**

Trigeminal ganglia (TG) neurons are an important site of lifelong latent varicella-zoster virus (VZV) infection. Although VZV-specific T-cells are considered pivotal to control virus reactivation, their protective role at the site of latency remains uncharacterized.

**Methods:**

Paired blood and TG specimens were obtained from ten latent VZV-infected adults, of which nine were co-infected with herpes simplex virus type 1 (HSV-1). Short-term TG-derived T-cell lines (TG-TCL), generated by mitogenic stimulation of TG-derived T-cells, were probed for HSV-1- and VZV-specific T-cells using flow cytometry. We also performed VZV proteome-wide screening of TG-TCL to determine the fine antigenic specificity of VZV reactive T-cells. Finally, the relationship between T-cells and latent HSV-1 and VZV infections in TG was analyzed by reverse transcription quantitative PCR (RT-qPCR) and in situ analysis for T-cell proteins and latent viral transcripts.

**Results:**

VZV proteome-wide analysis of ten TG-TCL identified two VZV antigens recognized by CD8 T-cells in two separate subjects. The first was an HSV-1/VZV cross-reactive CD8 T-cell epitope, whereas the second TG harbored CD8 T-cells reactive with VZV specifically and not the homologous peptide in HSV-1. In silico analysis showed that HSV-1/VZV cross reactivity of TG-derived CD8 T-cells reactive with ten previously identified HSV-1 epitopes was unlikely, suggesting that HSV-1/VZV cross-reactive T-cells are not a common feature in dually infected TG. Finally, no association was detected between T-cell infiltration and VZV latency transcript abundance in TG by RT-qPCR or in situ analyses.

**Conclusions:**

The low presence of VZV- compared to HSV-1-specific CD8 T-cells in human TG suggests that VZV reactive CD8 T-cells play a limited role in maintaining VZV latency.

**Supplementary Information:**

The online version contains supplementary material available at 10.1186/s12974-023-02820-y.

## Introduction

Most adults worldwide are infected with the neurotropic human alphaherpesvirus varicella-zoster virus (VZV), which causes chickenpox during primary infection and herpes zoster (HZ) in one-third of non-vaccinated individuals upon reactivation of latent VZV. Following primary infection, VZV establishes lifelong latency in neurons of trigeminal ganglia (TG) and dorsal root ganglia (DRG) along the entire neuraxis [1]. Furthermore, the live-attenuated VZV vaccine currently approved in many countries around the world has recently been reported to induce meningitis upon reactivation in a subset of vaccinated individuals [2]. The only signature of viral activity in latent VZV-infected neurons is the expression of three unique viral transcripts: VZV latency-associated transcript (VLT) and low levels of two isoforms of a fusion transcript containing VLT joined to gene 63 (VLT-ORF63) [3, 4]. While the VLT and VLT-ORF63-encoded proteins pVLT and pVLT-ORF63, respectively, are detected during lytic infection, no VZV proteins are detectable in latent VZV-infected human ganglia [5].

Whereas both innate and adaptive immune responses are crucial to control primary VZV infection, VZV-specific T-cells in particular are essential to prevent HZ [6, 7]. For example, individuals with disease- or therapy-induced T-cell deficiencies or elderly individuals with age-related declines in VZV-specific T-cell frequencies in blood are at increased risk of developing HZ [8]. In contrast, humoral immune defects have only limited effect on HZ incidence [6, 9]. A protective role of natural killer (NK) cell and virus-specific T-cell responses has also been reported for the closely related herpes simplex virus (HSV) [10, 11]. Previous studies in humans and mice have shown that T-cells, mainly CD8 T-cells, control HSV not only at the site of lytic infection (e.g., in the skin and mucosa) but also at the site of latency in ganglia [10, 12, 13]. At both of these peripheral sites, virus-specific T-cells are retained as tissue-resident memory T-cells (T_RM_) to control HSV reactivation [13, 14]. Although analogous infiltration and retention of virus-specific T_RM_ has been observed in the skin of VZV-infected individuals [15, 16], prior studies using only a small number of VZV proteins failed to detect VZV reactive T-cells in human TG [17]. However, this previous work was based on the prior assumption that a limited subset of VZV proteins is expressed by latently VZV-infected neurons [17]. More recent work has shown that this viral protein detection was artefactual [5, 18]. Thus, the aim of the present study was to unambiguously determine the presence and fine antigenic specificity of VZV reactive CD8 T-cells in latently VZV-infected human TG by performing an antigenic screen incorporating the entire VZV latent and lytic proteome, including recently identified protein pVLT, for ten deceased VZV latently infected adults.

## Materials and methods

### Human clinical specimens

Paired TG and heparinized blood samples were collected from ten deceased brain donors by the Netherlands Brain Bank (NBB; Amsterdam, The Netherlands; Additional file 3: Table S1). The NBB obtained written informed consent for brain autopsy as well as the use of clinical specimens and clinical information for research purposes from all study participants in advance. All procedures of the NBB have been approved by the Ethics Committee of Amsterdam University Medical Center (VUmc, Amsterdam, The Netherlands; project number 2009/148) and are in accordance with the Declaration of Helsinki. Formalin-fixed paraffin embedded (FFPE) TG biopsies from a separate cohort of five latent VZV-infected NBB brain donors were included for histological analyses (Additional file 4: Table S2). HSV-1 and VZV infection status of TGs was determined by (1) virus-specific ELISAs (Focus Technologies) on donor-derived plasma and/or (2) quantitative real-time PCR (qPCR) on DNA and cDNA obtained from TG cell suspensions to detect viral genomic DNA or latent HSV-1 latency-associated transcript (*LAT*) and VZV *VLT* RNA [3]. Infection status was scored as positive if either or both of these tests yielded a positive result.

### Generation of B- and T-cell lines

Human TG were enzymatically and mechanically dispersed using Liberase blendzyme 3 (0.2 units/mL, Roche) and GentleMACS (Miltenyi Biotech) to generate short-term TG-TCL as previously described [17]. Following Percoll-gradient-based lymphocyte enrichment, TG cell suspensions were stimulated with 1 µg/mL phytohemagglutinin-L (PHA-L; Roche) for 10–14 days in T-cell medium (TCM; RPMI-1640 supplemented with 10% heat-inactivated pooled human serum, 2 mM glutamine, and penicillin–streptomycin [Pen-Strep, Gibco]) in the presence of y-irradiated (3000 rad) allogeneic peripheral blood mononuclear cells (PBMC) and recombinant human interleukin 2 (Proleukin; rIL-2, 20 IU/mL) [19]. T-cells were further expanded using one or more rounds of anti-CD3 antibody (OKT3, Sanquin) treatment for 10–14 days in TCM supplemented with 20 IU/mL rIL-2 and the resultant TG-TCL were cryopreserved until use [16, 19]. Donor-derived PBMCs were incubated with Epstein–Barr virus (strain B95.8) to generate immortalized B-cell lines (B-LCL) that were used as autologous antigen presenting cells [19]. All cell cultures and functional T-cell assays were performed at 37 °C in a humidified CO_2_ incubator. TG-TCL from four donors have also been used in a previous study: TG01 (previously TG1), TG02 (previously TG2), TG03 (previously TG7), and TG04 (previously TG8) [19].

### Antigen-specific T-cell assays

T-cell reactivity to HSV-1 was determined by intracellular interferon gamma (IFNγ) staining of 100,000 TG-TCL cells co-incubated with 100,000 HSV-1 or mock-infected autologous B-LCL in TCM [19]. VZV reactivity was similarly determined using B-LCL pulsed overnight with a predefined dilution of clarified cell lysates of mock- or VZV-infected human retinal cells (ARPE-19 cells; ATCC CRL-2302) as described previously [20]. Cells were co-cultured for 6 h in TCM in the presence of monensin (Golgistop, BD Biosciences) to maintain IFNγ intracellularly, and stained with fluorochrome-conjugated mouse monoclonal antibody (mAb) to human CD3 (APC-Cy7, SP34-2, BD Biosciences), CD4 (Pacific Blue, RPA-T4, BD Biosciences), CD8α (PE-Cy7, RPA-T8, eBioscience), and for viability (LIVE/DEAD Fixable Aqua Dead Cell Stain Kit; ThermoFisher Scientific). Subsequently, cells were fixed and permeabilized using the Fixation/Permeabilization Kit (BD Biosciences), stained with a mAb antibody to IFNy (APC, B27, BD Biosciences), washed extensively, and analyzed on a BD FACS Lyric flow cytometer. 5000 to 25,000 viable CD3^+^ T-cells were acquired per sample and data were processed using FlowJo v10 software (BD Biosciences). Single, live CD3^+^ lymphocytes expressing either CD4 or CD8 were assessed for IFNy expression. TG-TCL stimulated with phorbol myristate–acetate (PMA; 50 ng/mL) and ionomycin (Iono; 500 ng/mL; both Sigma) served as positive control for overall T-cell reactivity.

Fine VZV T-cell specificity was determined by measuring IFNγ levels by ELISA (Invitrogen) in conditioned medium from 50,000 to 100,000 TG-TCL co-cultured for 24 h in TCM with artificial antigen-presenting cells (aAPC). As aAPC the African green monkey (*Chlorocebus aethiops*) cell line Cos-7 (ATCC CRL-1651) was used following co-transfection with up to four pcDNA3 vectors encoding the TG-donor’s specific HLA-A and -B alleles along with each of the canonical VZV open reading frames (ORFs) separately cloned into pDEST103, as described previously (15,000 cells per well) [16, 21]. To map the epitope within VZV ORF29, seven partially overlapping fragments were cloned into pDEST103 (Additional file 5: Table S3) and were sequence-confirmed. Stimulation with PHA-P (Remel, 1.6 µg/mL) served as positive control and the negative controls were Cos-7 cells transfected with HLA class I (HLA-I) cDNA only, with HLA-I and empty pDEST103 vector, and non-transfected cells. TG-TCL were defined as VZV ORF reactive if both replicates exceeded IFNy ELISA OD_450_ values of 0.15 and were at least twice the value of the pDEST103 empty vector negative control. Reactive TG-TCL were analyzed in-depth to map the cognate epitope as previously described [16]. Briefly, Cos-7 cells transfected with the respective HLA-I cDNA for 48–72 h were combined overnight with TG-TCL in the presence of 1 μg/mL synthetic peptide in TCM. Peptides were reconstituted in DMSO before use and DMSO-only was used as negative control, while transfection with the full-length VZV ORF or PHA-P treatment were used as positive controls. Peptide pools spanning VZV ORF9 consisted of 13-mers overlapping by 9-amino acids (Genscript). Single peptides were designed using NetMHCpan EL4.1 algorithmic prediction of binding to the restricting HLA-I allele or according to ORF homology between HSV-1 (strain 17; Genbank Accession number NC_001806) and VZV (strain Dumas; Genbank Accession number X04370) [22].

### Real-time qPCR analysis

DNA and RNA extraction from human TG samples and subsequent cDNA synthesis were performed as described previously [3, 17]. Real-time qPCR analysis was performed on an ABI Prism 7500 Real-Time PCR system using 4 × Taqman Fast Advanced Master mix (Applied Biosystems) and primers and probes specific for HSV-1 *UL38* [17], VZV *ORF38* [17], HSV-1 *LAT* [17], VZV *VLT* [3], and *GAPDH* [23]. In addition, 18 TGs (clinical characteristics described in [3]) were analyzed for VZV DNA, VZV transcript *VLT,* or fusion transcript *VLT-ORF63* in combination with *CD3D* (Hs00174158_m1; ThermoFisher) transcript levels. Abundance of target genes was calculated by normalizing for cellular housekeeping gene *GAPDH* [relative expression = 2^ − ((average Ct-value *CD3D*) − (average Ct-value *GAPDH*))].

### Sequence alignment and peptide–HLA binding prediction

Geneious Prime version 2022.2.2 (Dotmatics) and the Geneious alignment algorithm with default parameters were used to align viral sequences. The predicted binding rank for minimal HSV epitopes and their VZV homologous peptides of similar length to relevant HLA-I alleles was analyzed using the netMHCpan4.1 algorithm with default parameters accessed at IEDB [22].

### In situ analysis

Consecutive 5 µm-thick FFPE TG sections from five latent VZV-infected subjects (Additional file 4: Table S2) were analyzed for CD3 protein expression and VZV *VLT* RNA expression by immunohistochemistry (IHC) and in situ hybridization (ISH), respectively, as described previously [3, 24]. In brief, IHC was performed using mouse anti-CD3 antibody (clone F7.2.38), biotin-conjugated goat anti-mouse IgG antibody, and horseradish peroxidase-conjugated streptavidin (all from ThermoFisher). Staining was visualized using 3-amino-9-ethylcarbazole (AEC) and sections were counterstained with hematoxylin (Sigma-Aldrich). ISH was performed using probes specific to VZV *VLT* [3], mRNA of the human gene *POLR2A* (encoding DNA-directed RNA polymerase II subunit RPB1; positive control), and the bacterial gene *dapB* (encoding 4-hydroxy-tetrahydrodipicolinate reductase, Genbank Accession number EF191515; negative control) using the RNAScope 2.5 HD Assay (Advanced Cell Diagnostics). RNA integrity in tissue sections and specificity of the RNAScope Assay was demonstrated by robust *POLR2A* staining and absence of *dapB* ISH signal in all sections analyzed (Additional file 1: Figure S1). Complete tissue sections were scanned using the Nanozoomer 2.0 HT (Hamamatsu) and analyzed using NDP.view2 (Hamamatsu).

### Statistical analysis

Statistical analysis was performed using GraphPad Prism (version 9; Graphpad Software). Data were analyzed using two-tailed Spearman exact test where appropriate. Results were considered significant at *p* < 0.05.

## Results

### Detection of VZV reactive T-cells in latently infected TG

**Fig. 1:**
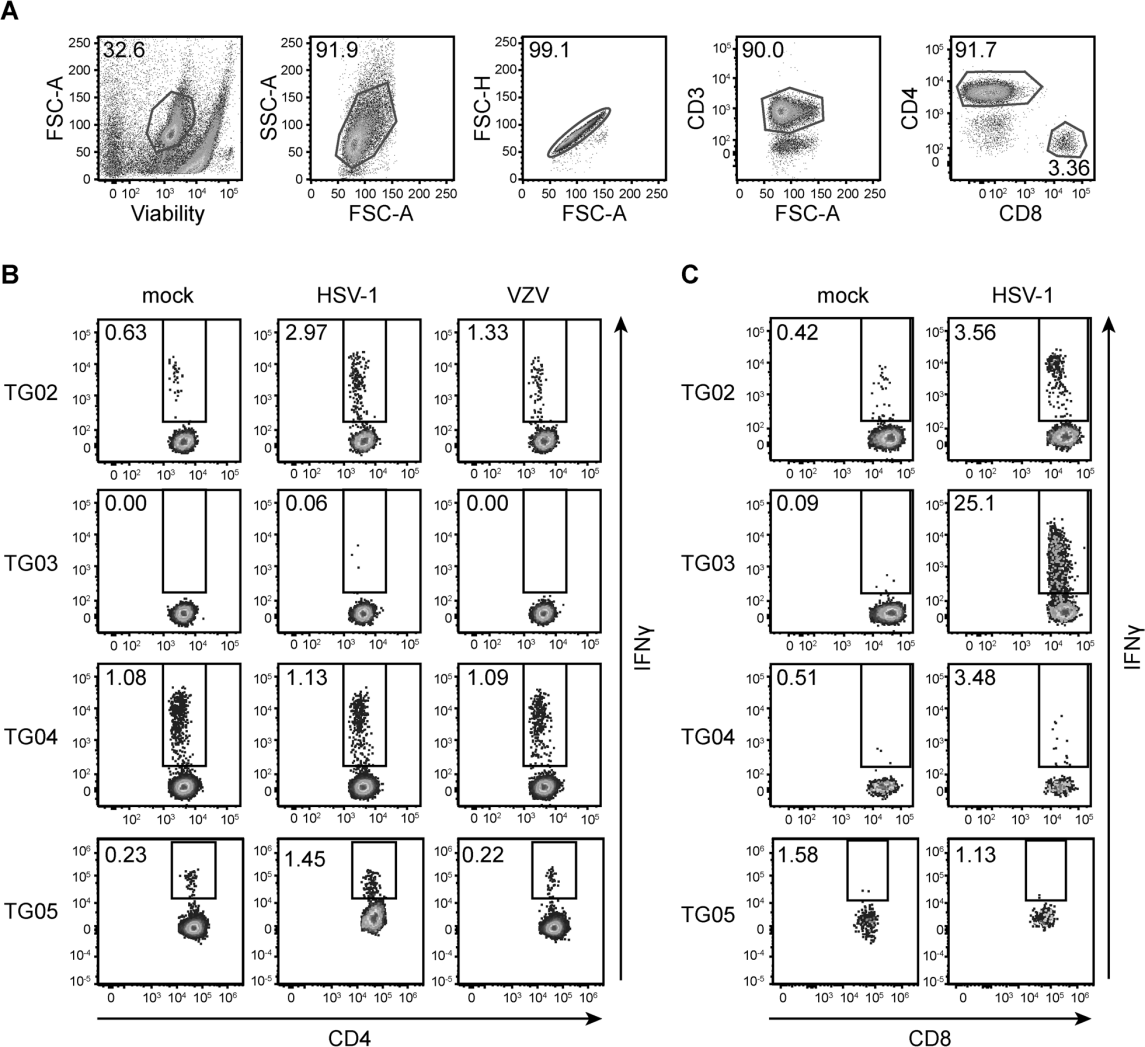
HSV-1 and VZV responses of human TG-derived T-cell lines. **A** Gating strategy applied to determine T-cell reactivity, consisting of selection of viable cells followed by size exclusion and selection of single cells, selection of CD3^+^ T-cells, and finally selection of CD3^+^CD4^+^ and CD3^+^CD8^+^ T-cells. FSC-A, forward scatter—area; SSC-A, side scatter—area; FSC-H, forward scatter—height. **B** Flow cytometry analysis of IFNγ production by CD4 T-cells derived from TG donors TG02–TG05 following 6 h of co-culture with autologous B-LCL pulsed with lysates derived from mock, HSV-1, or VZV-infected ARPE-19 cells. **C** Flow cytometry analysis of IFNγ production by CD8 T-cells following 6 h of coculture with autologous mock- or HSV-1-infected B-LCL. Numbers indicate percentages of cells within the respective gate

Heparinized blood and TG were obtained during autopsy from ten individuals living in The Netherlands with an average ± standard deviation (SD) post-mortem interval of 6 ± 1.5 h. The cohort included 2 men and 8 women, the average age was 80 ± 11 years, and the majority had an underlying neurodegenerative disease, such as Alzheimer’s disease or multiple sclerosis. The cause of death was not related to acute herpesvirus infections (Additional file 3: Table S1). Nine subjects were latently infected with both HSV-1 and VZV, one was infected with VZV only (subject TG07; Additional file 3: Table S1). Short-term TG-TCL were successfully generated from all subjects by mitogenic stimulation of T-cells recovered from the TG. Autologous B-LCL were generated from PBMC of 4 of the 10 individuals (TG02–TG05). For the other six persons B-LCL generation failed due to low viability of the PBMC isolated from post-mortem blood samples. To investigate whether VZV reactive T-cells are present in human TG, analogous to HSV-1 reactive T-cells that were found in previous studies [10, 11], we first analyzed whole VZV and HSV-1-specific T-cell reactivity in these four donors. To examine CD4 T-cell reactivity, exogenous antigen was provided to the B-LCL by pulsing with predefined concentrations of UV-irradiated lysates of mock, HSV-1-, or VZV-infected ARPE-19 cells. To provide endogenous antigens and evaluate CD8 T-cell activation, B-LCL were infected with HSV-1. Since B-LCL do not support productive VZV infection, CD8 T-cell reactivity to VZV was separately evaluated using a different method as described below. Consistent with our earlier studies [10], robust HSV-1-specific CD8 T-cell reactivity and, to a lesser degree, CD4 T-cell reactivity were observed in response to HSV-1-infected autologous B-LCL (Fig. 1 and Table 1). In contrast, we observed low frequencies of VZV reactive CD4 T-cells in only one of the four TG-TCL, suggesting that VZV-specific CD4 T-cell prevalence was much lower than for HSV-1.

**Table 1:** Virus-specific T-cell reactivity in TG-derived T-cell lines

	T-cell reactivity (%)*			
Subject	HSV-1		VZV	
ID	CD4	CD8	CD4	CD8
TG02	2.5	3.2	0.7	NA
TG03	0.1	27.0	0.0	NA
TG04	0.1	4.0	0.0	NA
TG05	1.3	0.0	0.0	NA

*Net percentages of virus reactive T-cells in human TG-derived T-cell lines, as determined by flow cytometric detection of intracellular IFNγ expression following coculture with autologous B-LCL infected with HSV-1 (for CD8) or pulsed with HSV-1 or VZV lysate (for CD4). T-cell reactivities to mock-infected B-LCL were subtracted. NA, not applicable as B-LCL pulsed with exogenous antigens do not efficiently present antigens to CD8 T-cells

### VZV proteome-wide screening of CD8 T-cells recovered from human TG

**Fig. 2:**
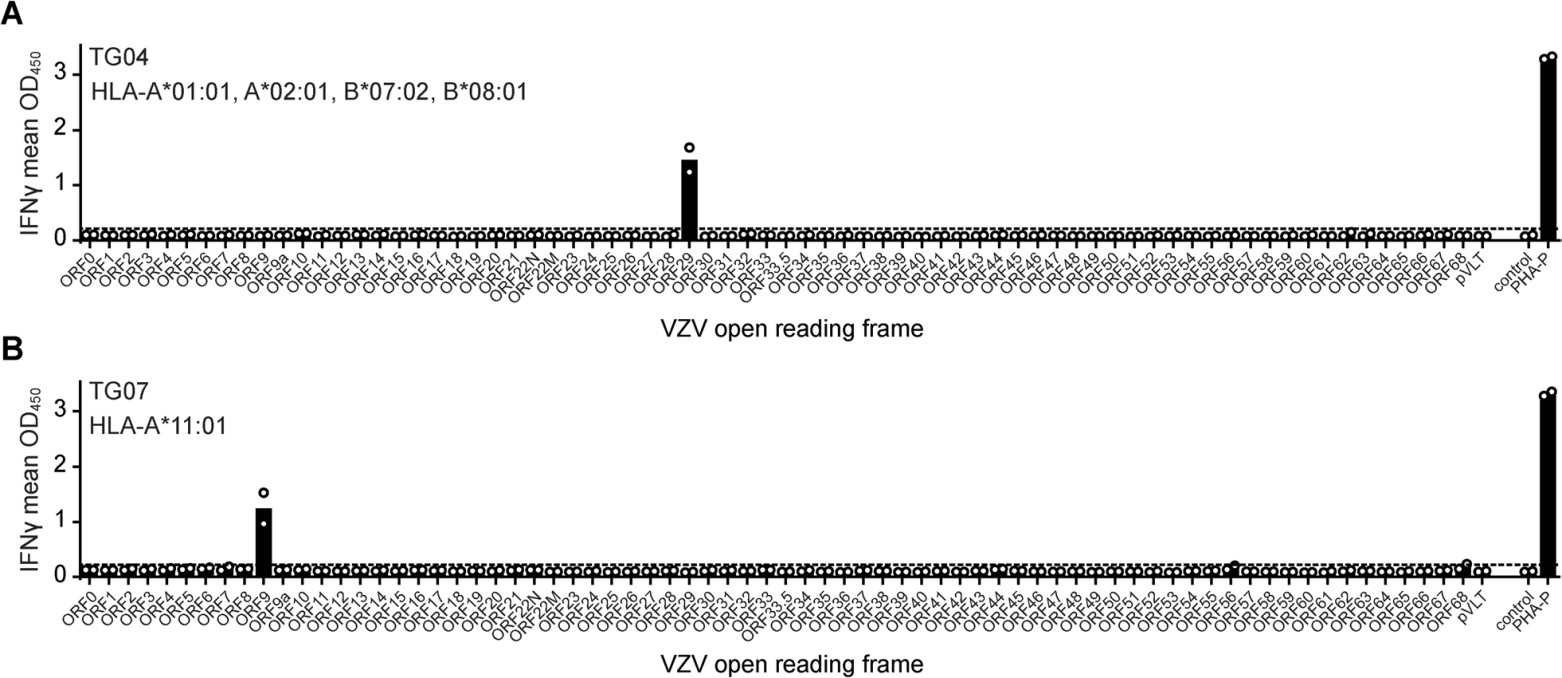
VZV antigen specific response of human TG-derived CD8 T-cells. **A, B** TG-derived T-cell lines of donor TG04 (**A**) or TG07 (**B**) were screened for reactivity with artificial antigen presenting cells expressing the indicated subject-specific HLA class I allele(s) and individual VZV open reading frames or fragments thereof. For subject TG04, all 4 HLA alleles were co-transfected simultaneously, while for subject TG07, only HLA-A*11:01 was used. Empty vector (control) and phytohemagglutinin (PHA-P), were used as negative and positive controls, respectively. Levels of secreted IFNγ was determined by ELISA. Data are presented as the individual (circles) and mean (bars) OD_450_ values of two independent replicates. Horizontal dashed lines indicate the threshold of T-cell response set at twice the value of the empty vector control. Viral ORF nomenclature according to VZV reference strain Dumas (Genbank Accession number X04370)

T-cells are considered instrumental in controlling HSV-1 latency. Specifically CD8 T-cells are the most abundant T-cell subset in latently HSV-1-infected human and mouse ganglia [10]. As mentioned, the assessment of CD8 T-cell reactivity to VZV using the B-LCL coculture method was impeded by the fact that B-LCL do not support productive VZV infection (data not shown). Therefore, we performed VZV genome-wide screens to assess the presence and fine antigen specificity of VZV reactive CD8 T-cells in the 10 TG-TCL (TG01–TG10) using aAPC. Cos-7 cells were co-transfected with (1) subject-specific HLA-A and -B cDNA (to a maximum of 4 alleles per subject) together with (2) individual viral genes that together encompass the complete VZV proteome, including pVLT, for use as aAPC [23, 25]. In this system, the β2-microglobulin co-factor required for HLA-I is endogenously expressed by the Cos-7 cells. We then assayed the respective short-term TG-TCL for IFNγ secretion in response to incubation with the HLA-I matched aAPC. We have previously applied this platform to uncover the targets and diversity of HSV- and VZV-specific T-cells in human blood and other tissues in an unbiased way [16, 19, 25–27]. While all TG-TCL responded strongly to the control mitogen PHA-P, demonstrating their ability to secrete IFNγ, only 2 of 10 TG-TCL showed VZV-protein specific CD8 T-cell reactivity: the TG-TCL of subject TG04 and TG07 responded to VZV ORF29 and ORF9, respectively (Fig. 2 and Additional file 2: Figure S2). Together, the data indicate that VZV reactive CD8 T-cells are scarce in human latently VZV-infected TG.

### Epitope mapping of VZV reactive CD8 T-cells recovered from human TG

Next, the fine antigenic specificity of the VZV reactive CD8 T-cells in subjects TG07 and TG04 was defined by analyses using protein fragments and synthetic peptides.

**Fig. 3:**
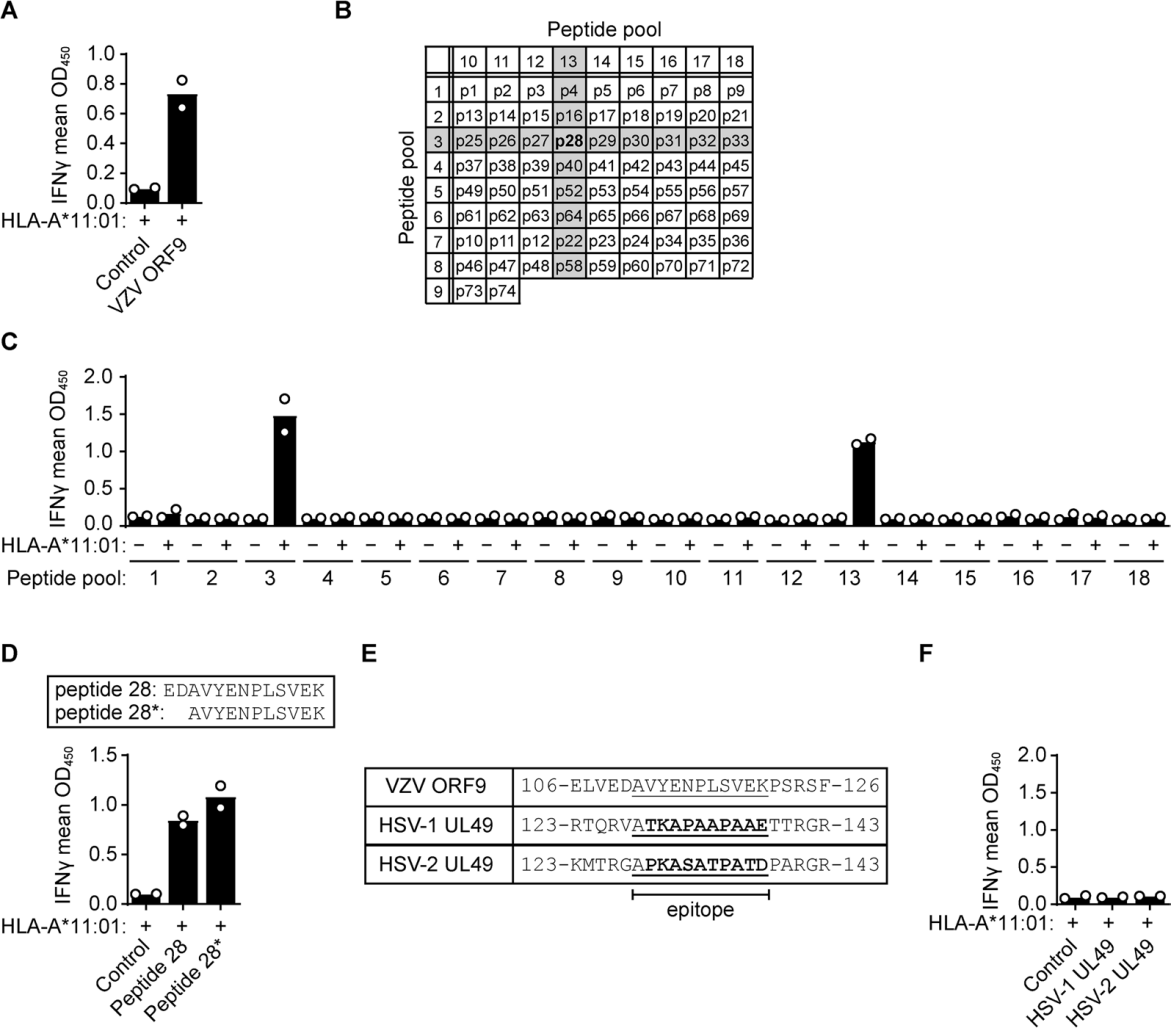
TG-derived CD8 T-cells recognize an epitope in VZV ORF9 epitope in an HLA-A*11:01-dependent manner. **A** IFNγ secretion by TG07-derived T-cells following 24 h of incubation with Cos-7 cells co-transfected with a vector encoding HLA-A*11:01 and either an empty control vector or a vector encoding VZV ORF9. **B** Overview of the peptide composition of each ORF9 peptide pool used in panel C. Reactive pools are shaded. **C** IFNγ secretion by TG07-derived T-cells co-cultured with Cos-7 cells transfected with HLA-A*11:01 and pulsed with the indicated peptide pools, showing reactivity for two individual pools that overlap at peptide 28. **D** IFNγ secretion by TG07-derived T-cells following co-culture with HLA-A*11:01-transfected Cos-7 cells transfected and pulsed with ORF9 peptide 28 (amino acids 109–121, EDAVYENPLSVEK) or internal ORF9 peptide 28* (amino acids 111–121, AVYENPLSVEK). **E** Alignment of the VZV ORF9 epitope (AVYENPLSVEK, underlined) with the homologous regions of HSV-1 and HSV-2 UL49. Amino acids in bold differ between VZV and HSV-1/HSV-2; numbers indicate amino acid positions in the respective proteins. **F** IFNγ secretion by TG07-derived T-cells following co-culture with Cos-7 cells transfected with HLA-A*11:01 in combination with either an empty control vector or vectors encoding HSV-1 UL49 or HSV-2 UL49. Data are presented as the individual (circles) and mean (bars) OD_450_ values of at two independent replicates

We found that the ORF9 CD8 T-cell response of subject TG07 was mediated by HLA-A*11:01 (Figs. 2B and 3A). Using synthetic peptide pools that together completely covered VZV ORF9, we narrowed down the epitope to residues 109–121 of ORF9 (peptide 28, EDAVYENPLSVEK) (Fig. 3B, C). Further truncation of the peptide identified the minimal epitope corresponding to amino acids 111–121 of ORF9 (AVYENPLSVEK; Fig. 3D). HSV-1/HSV-2 and even HSV/VZV cross-reactive B- and T-cell responses have been observed due to the extensive homology between the proteins of these viruses [25, 28, 29]. However, in silico analysis of the CD8 epitope-containing region of VZV ORF9 showed that there is little amino acid homology to the UL49 protein orthologues in HSV-1 or HSV-2 (Fig. 3E). In line with this, no IFNy secretion by the TG-TCL of subject TG07 was observed in response to Cos-7 cells expressing HLA-A*11:01 and either HSV-1 or HSV-2 full-length UL49 protein (Fig. 3F). These data demonstrate that the ORF9 reactive CD8 T-cell response was solely VZV specific. This conclusion is further supported by the HSV-1 seronegative status of this subject (Additional file 3: Table S1).

**Fig. 4:**
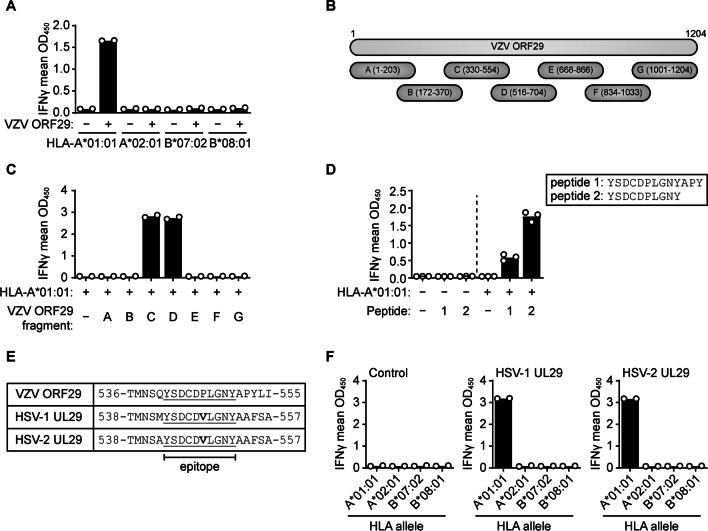
TG-VZV reactive CD8 T-cells recognize an HSV/VZV cross-reactive epitope in an HLA-A*01:01-dependent manner. **A** IFNγ secretion by TG04-derived T-cells following incubation with Cos-7 cells transfected with the indicated subject-specific HLA class I allele together with an empty control vector (−) or a vector encoding VZV ORF29 (+). **B** Schematic of VZV ORF29 fragments used in (**C**) to map the T-cell epitope. Numbers indicate amino acid positions. **C** ELISA showing IFNγ secretion by TG04-derived T-cells following incubation with Cos-7 cells transfected with HLA-A*01:01 and an empty control vector (−) or vectors encoding the indicated VZV ORF29 fragments from (**B**). **D** IFNγ production by TG04-derived T-cells following coculture with Cos-7 cells transfected without (−) or with (+) HLA-A*01:01 and pulsed with the indicated ORF29 peptides or DMSO as a negative control (−). **E** Alignment of the VZV ORF29 epitope-containing region of VZV ORF29 with the homologous regions of HSV-1 UL29 and HSV-2 UL29 (underlined). Amino acid in bold differ between VZV, HSV-1, and HSV-2. Numbers indicate amino acid positions in the respective proteins. **F** IFNγ secretion by TG04-derived T-cells following co-culture with Cos-7 cells transfected to express the indicated HLA class I alleles in combination with either an empty control vector or vectors encoding full-length HSV-1 UL29 or HSV-2 UL29. Data are presented as the individual (circles) and mean (bars) OD_450_ values of 2 (**A, C, F**) or 3 (**D**) independent replicates

In subject TG04, the ORF29 CD8 T-cell response was HLA-A*01:01-restricted (Fig. 4A). Using ORF29 fragments we mapped the epitope to the region overlapping between sections C and D, containing amino acids 516 to 554 (Fig. 4B, C). Subsequent synthetic peptide mapping localized the epitope to ORF29 residues 541–553 and then to 541–550 (YSDCDPLGNY) (Fig. 4D). Notably, this CD8 T-cell epitope is located in a region of VZV ORF29 showing extensive homology with its HSV-1 and HSV-2 orthologous protein UL29 (Fig. 4E). Recognition of both HSV-1- and HSV-2 UL29-transfected Cos-7 cells expressing HLA-A*01:01 functionally confirmed cross-reactivity (Fig. 4F), thereby identifying this ORF29 epitope as a human alphaherpesvirus cross-reactive CD8 T-cell epitope. Notably, subject TG04 was seropositive for both HSV-1 and VZV (Additional file 3: Table S1). Taken together, VZV-specific T-cell immunity in human TG was composed of both VZV unique (TG07) and human alphaherpesvirus cross-reactive (TG04) T-cell responses.

### Human alpha-herpesvirus cross-reactive CD8 T-cells are rare in HSV-1/VZV co-infected human TG

Since we found that one of the two identified VZV epitopes was cross-reactive between HSV and VZV, we explored the potential for HSV/VZV cross-reactive CD8 T-cell responses in human TG in more detail. Using an extended set of 10 HSV-1 epitopes previously found to be recognized by CD8 T-cells from human TG latently infected with both HSV-1 and VZV [19], we compared the HSV-1 epitope-containing protein regions for homology with VZV using sequence alignment and HLA-I binding prediction software. Of these 10 HSV-1 CD8 T-cell epitopes, two did not show any amino acid homology in the corresponding VZV protein (epitopes 1 and 10; Additional file 6: Table S4). For the remaining 8 epitopes, homologous regions in the corresponding VZV protein could be identified (epitopes 2–9). For HSV-1 epitopes 2, 3, and 5, the VZV homologs had very poor predicted binding ranks to HLA-I (Additional file 6: Table S4). For the remaining epitopes (4, 6, 7, 8, and 9), the VZV homolog peptide did show relatively preserved predicted binding to the relevant HLA-I allele. However, we observed 4 to 7 amino acid differences among the total epitope length of 9 to 10 residues between the proven HSV-1 epitope and the VZV homolog. Thus, despite the potential HLA binding, these VZV homologous peptides are highly unlikely to induce activation of the HSV-1-specific TCR due to the extensive amino acid mismatches. These data suggest that alphaherpesvirus cross-reactive CD8 T-cells are uncommon in latently HSV-1/VZV co-infected human TG.

### No association between T-cell infiltrates and VZV latency in human TG

**Fig. 5:**
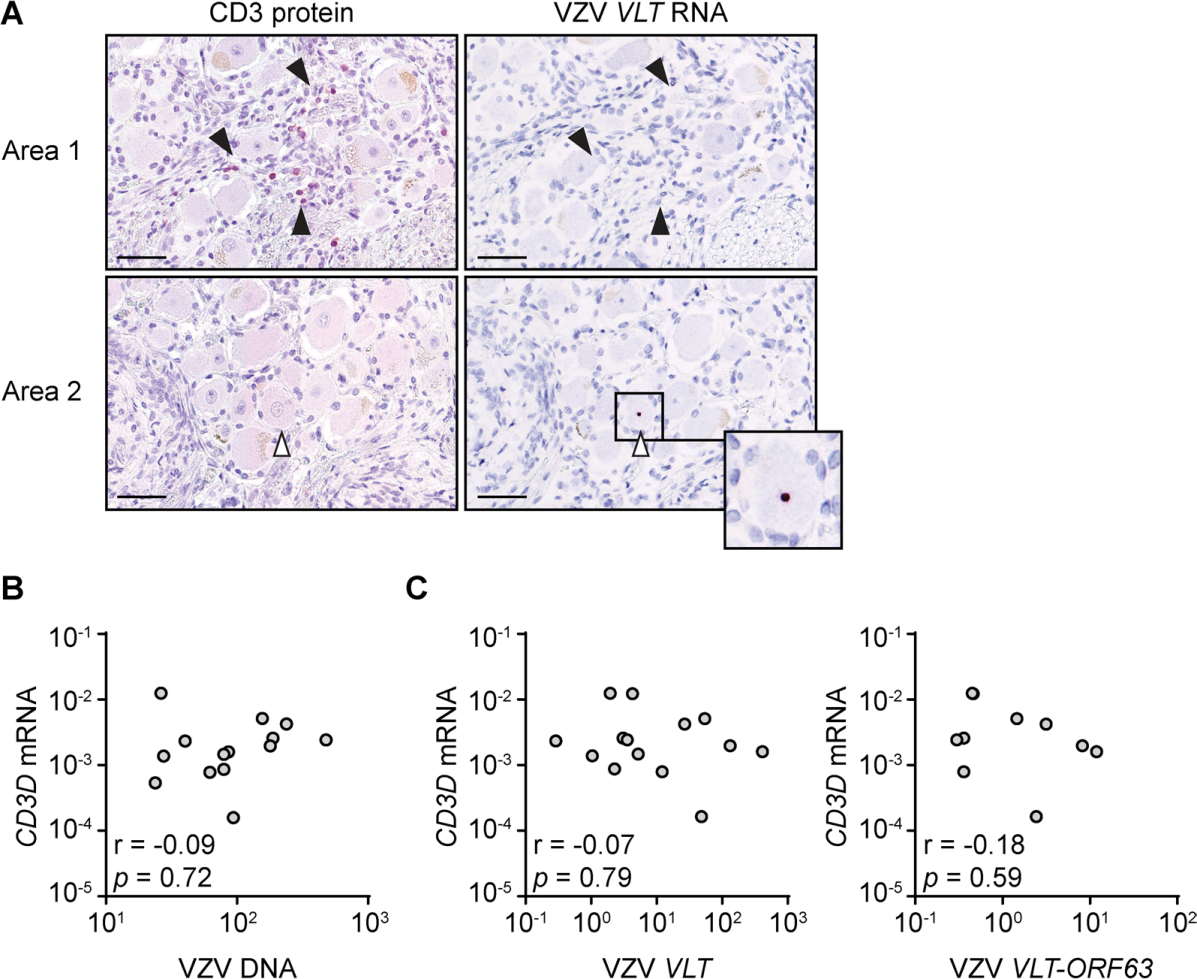
No correlation between T-cell infiltrates and VZV latency in human TG. **A** Consecutive TG sections were stained for CD3 by immunohistochemistry (red; left column) or *VLT* RNA by in situ hybridization (dark red, right column). Filled and open arrow heads indicate CD3^+^ cells and a *VLT*^+^ neuron, respectively; the inset shows a *VLT*^+^ neuron. Data is shown for two different areas (1 and 2) of one donor that are representative of five independent TG donors; scale bar: 50 µm. **B, C** Abundance of VZV DNA (**B**) or VZV *VLT* and VZV *VLT*-*ORF63* RNA (**C**) as well as human *CD3D* transcripts in 18 TG specimens (described in [3]) as determined by q(RT)-PCR and presented as relative DNA/transcript levels normalized to single-copy gene *HMBS* or cellular housekeeping gene *GAPDH*. Spearman *r* and *p* values are indicated

Our findings imply that VZV-specific CD4 and CD8 T-cells are rare in human TG and found in fewer individuals compared to HSV-1-specific T-cells. To corroborate this finding, we performed two alternative independent assays on human TG. First, we examined the spatial orientation of T-cells in relation to latently infected TG neurons. We and others have previously observed T-cell clusters in close proximity to latent HSV-1-infected (*LAT*^+^) neurons in human and mouse ganglia [13, 19]. Here, we determined CD3 and *VLT* expression in consecutive TG sections (*n* = 3 per donor) from five VZV-infected subjects by IHC and ISH (Additional file 4: Table S2). In total, we analyzed 16,140 neurons (on average 1,076 per section), of which 0.36% (SD: 0.18%) expressed *VLT*. Although sparse T-cell accumulations were present in some areas of the TGs (Fig. 5, area 1), these were not observed around *VLT*-expressing, latently VZV-infected neurons (area 2). Second, we have previously observed that the level of *CD8α* transcripts—a measure for T-cell infiltration/retention—correlates with HSV-1 genome load in human TG, indicating that the extent of T-cell infiltration into the TG correlates with HSV-1 viral load [19]. Here, we similarly determined the correlation between the abundance of *CD3D* T-cell transcripts and VZV DNA or viral RNAs selectively expressed during VZV latency (*VLT* and the *VLT*-*ORF63* fusion transcript) [3, 4, 19]. No correlation was observed between VZV DNA, VZV *VLT*, or VZV *VLT*-*ORF63* RNA levels and *CD3D* transcript abundance, suggesting that T-cell infiltration and/or retention in the human TG does not correlate with VZV viral load (Fig. 5B, C). Together with the functional T-cell data, these data support that retention of VZV-specific T-cells is limited in latent VZV-infected human TG.

## Discussion

Previous studies have shown that local virus-specific T-cell responses are instrumental to control HSV infections, both at sites of lytic (e.g., the skin and mucosal tissues) as well as latent infection (e.g., the TG). Whereas VZV-specific T-cells are known to be protective against HZ, their localization and functional roles at the sites of viral latency remain unknown. In the present report, we assessed the abundance of VZV reactive T-cells in human TGs by determining the reactivity of T-cells derived from latently VZV-infected TG of ten elderly adults. Our data show that VZV reactive CD8 T-cells are present in only a small subset of latently VZV-infected human TG and that they are much less frequently observed than HSV-1-specific T-cells.

We used two complementary APC platforms to detect virus-specific T-cells in human TG-TCL. First, we cocultured the TG-TCL with autologous B-LCL as APC and observed robust frequencies of HSV-1 reactive T-cells, especially CD8 T-cells, in 3 of 4 TG-TCL (75%). This is in line with the selective retention of HSV-1-specific T-cells in human TG latently infected with HSV-1 that we have previously observed [17]. In contrast, CD4 T-cell reactivity towards B-LCL pulsed with VZV antigens was detected in only 1 of the 4 TG-TCL tested (25%). The reactivity of CD8 T-cells to VZV was probed more extensively, using our aAPC system based on Cos-7 cells that were co-transfected with the TG-donor-specific HLA-A and -B alleles and individual components of the entire VZV proteome, including pVLT [16, 19, 26]. Only 2 VZV proteins were found to be antigenic in 2 of 10 TG-TCL (20%) in this unbiased comprehensive analysis. This is much lower than observed for HSV-1 in our previous study, which showed CD8 T-cell reactivity towards HSV-1 in 8 of 12 TG-TCL (67%), resulting in a total of 13 HSV-1 proteins targeted by CD8 T-cells [19]. Taken together, our data indicate low prevalence of VZV-specific CD4 and CD8 T-cells in human TG.

Of the two VZV epitopes we identified, the epitope in ORF29 was found to be cross-reactive with the homologous HSV-1/2 proteins UL29. This is in line with a previous study in which we also identified abundant cross-reactive T-cells targeting ORF29/UL29 in blood-derived T-cells [25]. In fact, 10–50% of blood-derived HSV-1 reactive CD4 and CD8 T-cells are HSV-1/VZV cross-reactive and HSV-1 proteins recognized by TG-resident CD8 T-cells such as ICP4 (VZV ORF62), ICP6 (VZV ORF19), and VP16 (VZV ORF10) are major contributors to HSV/VZV cross-reactive T-cell immunity [19, 25]. It is thus plausible that the UL29/ORF29 cross-reactive T-cells detected in this study are a reflection of this substantial level of cross-reactivity between VZV and HSV, and VZV may not have been the primary inducer and/or target of these T-cells in this HSV-1/VZV co-infected TG donor.

To determine how widespread the cross-reactivity of HSV-1 reactive T-cells with VZV antigens is, we performed an in silico analysis of 10 previously reported HSV-1 minimal epitopes [19]. Whereas for several epitopes the predicted HLA-binding score for the homologous VZV epitope was very low, for another subset the homologous VZV peptides regained good binding prediction scores for the relevant HLA-I allele (Additional file 6: Table S4). Nevertheless, even for these epitopes, it remains unlikely that T-cell receptors (TCR) that bind the ligand created by HSV-1 peptides and HLA-I will cross-react with VZV, since TCR are extremely polymorphic, with hypervariable CDR3 domains from both α and β chains contributing to binding to peptide/HLA-I complexes. The presence of 4 to 7 amino acid differences between the 9 to 10 amino acid HSV-1 epitopes and their VZV homologs strongly suggests that TCR cross-reactivity is unlikely [30, 31]. In support of this conclusion, we have previously reported that HSV-2-specific CD8 T-cells fail to recognize a variant peptide from HSV-2 strains that circulate widely in the general population that differs in only 2 of 9 residues, despite retained binding to the relevant HLA-I allele [32].

The other identified epitope that was specific to VZV mapped to ORF9. We recently reported that VZV ORF9 was highly immunoprevalent in human skin- and blood-derived TCL being frequently recognized by CD8 T-cells in these tissues [16]. In fact, the exact ORF9_111–121_ epitope found here was also identified as a CD8 T-cell epitope in skin-derived TCL from the site of a healed HZ lesion [16]. This suggests that ORF9 is a widely targeted VZV protein with an important role in controlling VZV infection, warranting the consideration of including ORF9 in future VZV subunit vaccine preparations.

All except one subjects in our study were HSV-1/VZV co-infected. Nevertheless, our current observations as well as prior studies indicate that the majority of alphaherpesvirus-specific T-cells in TG are directed to HSV-1 and not VZV [17, 19]. We detected robust HSV-1-specific CD8 T-cell responses in three of four donors (75%) analyzed (Table 1), but VZV reactive CD8 T-cells in only two of ten TG (20%) analyzed (Fig. 2 and Additional file 2: Figure S2). These findings are supported by the detection of abundant T-cells by immunohistology in HSV-1-infected TG, but not in TG solely infected with VZV [19, 33], as well as the lack of correlation between the abundance of T-cells and *VLT* expression in TG (Fig. 5). Moreover, a previous study reported the absence of T-cell infiltrates in human DRG that commonly contain latent VZV but not HSV-1 [34].

In contrast to the scarcity of VZV reactive T-cells in TG we observed here, we have recently reported the abundant presence and long-term persistence of virus-specific T_RM_ cells in HZ-affected skin sites, indicating that VZV-specific T-cells are functionally induced in VZV-infected individuals and are recruited to sites of lytic VZV infection [16]. The disparity in virus-specific T-cell retention between HSV-1 and VZV may thus be due to differences in their reactivation patterns. Whereas asymptomatic and symptomatic HSV-1 reactivation occurs frequently, asymptomatic VZV reactivation is thought to be rare and clinically apparent VZV reactivation typically occurs once in a lifetime in immunocompetent individuals [35, 36]. The low reactivation frequency of VZV may thus provide insufficient antigens for the long-term selective retention of VZV-specific T-cells in the TG. Alternatively, VZV reactivation in the TG may be controlled by other components of the immune system, for example, infiltrating innate immune cells and resident satellite glial cells. While neurons do not express MHC class II, the abundant neuron-interacting satellite glial cells in TG can potentially process and present antigen to infiltrating CD4 T-cells [37]. In support of this, we observed CD4 T-cell reactivity to VZV in 1 of 4 tested subjects (Table 1). However, since our current study focused on CD8 T-cells, the relevance of CD4 T-cells in controlling VZV infection will require further investigation.

The TG donors included here were of high age (average 80 ± 11 years), and therefore, potentially experienced waning VZV-specific T-cell immunity. VZV-specific memory T-cells are maintained at a low frequency in skin and blood throughout life, but decline in number and/or functional capacity with older age leading to increased risk of developing HZ [15, 38–41]. The low prevalence and abundance of VZV reactive T-cells detected may thus be an underestimate compared to young adults. Future studies in younger subjects and using alternative assays, including VZV protein-specific tetramer staining [16], to re-analyze VZV-specific T-cell memory in human TG may offer further insight. Because varicella and zoster vaccination is not recommended by the Dutch government, the VZV vaccination rates in The Netherlands are very low. Nonetheless, we cannot rule out that any of the TG donors have been vaccinated prior to death, which could have shaped the VZV-specific T-cell repertoire in the respective TG. Furthermore, we investigated TG specifically, since VZV reactivations are disproportionally common in the TG relative to DRG [42]. Nevertheless, VZV establishes latent infection in ganglia along the entire neuraxis and whether T-cell-mediated immunity plays a differential role in other ganglia than TG remains to be determined.

In conclusion, the current study shows that VZV-specific CD8 T-cells are scarce in latently VZV-infected human TG, suggesting that the protective role of VZV reactive CD8 T-cells against herpes zoster is of limited importance at the site of latency.

## Supplementary Information

**Additional file 1: Figure S1.** Control stainings for the in situ hybridization assays. Staining for the human gene *POLR2A *and the bacterial gene *dapB *were performed as positive and negative controls, respectively, for the in situ hybridization assays shown in main, Fig. 5; scale bar: 50 µm.

**Additional file 2: Figure S2.** VZV antigen-specific responses of additional human TG-derived CD8^+^ T-cells. TG-derived T-cell lines of donor TG01–TG03 and TG05–TG10 were incubated with Cos-7 cells transfected with vectors encoding the indicated subject-specific HLA class I allele together with the individual VZV open reading frames. Empty vector and phytohemagglutinin were used as negative and positive controls, respectively. Levels of secreted IFNγ were determined by ELISA. Data are presented as the individual and mean OD_450_ values of two independent replicates. Horizontal dashed lines indicate the threshold of T-cell response set at twice the value of the empty vector control. Viral ORF nomenclature according to VZV reference strain Dumas.

**Additional file 3: Table S1.** General characteristics and HLA genotype of study subjects used for T-cell analysis.

**Additional file 4: Table S2.** General characteristics of the study subjects used for histological analysis in main, Fig. 5.

**Additional file 5: Table S3.** Primers used to generate the VZV ORF29 truncated fragments.

**Additional file 6: Table S4.** Homology of HSV-1 CD8 T-cell epitope-containing regions with orthologous VZV proteins.

## Data Availability

The data sets used and/or analyzed during the current study are available from the corresponding authors upon reasonable request.

## Abbreviations

B-LCL: B-Lymphoblastoid cell line
DRG: Dorsal root ganglion
EBV: Epstein–Barr virus
FFPE: Formalin-fixed paraffin embedded
HLA-I: Human leukocyte antigen class I
HSV-1: Herpes simplex virus type 1
ICS: Intracellular cytokine staining
IFNγ: Interferon gamma
HZ: Herpes zoster
PBMC: Peripheral blood mononuclear cells
ORF: Open reading frame
PHA-L: Phytohemagglutinin-L
PHA-P: Phytohemagglutinin-P
RT-qPCR: Reverse transcription quantitative real-time PCR
TCL: T-cell line
TCM: T-cell medium
TCR: T-cell receptor
TG: Trigeminal ganglion
VZV: Varicella-zoster virus

## References

[1] Knipe DMPMH (2020). Field’s Virology.

[2] Ramachandran PS, Wilson MR, Catho G (2021). Meningitis caused by the live varicella vaccine virus: metagenomic next generation sequencing, immunology exome sequencing and cytokine multiplex profiling. Viruses.

[3] Depledge DP, Ouwendijk WJD, Sadaoka T, Braspenning SE, Mori Y, Cohrs RJ, Verjans GMGM, Breuer J (2018). A spliced latency-associated VZV transcript maps antisense to the viral transactivator gene. Nat Commun.

[4] Ouwendijk WJD, Depledge DP, Rajbhandari L, Lenac Rovis T, Jonjic S, Breuer J, Venkatesan A, Verjans GMGM, Sadaoka T (2020). Varicella-zoster virus VLT-ORF63 fusion transcript induces broad viral gene expression during reactivation from neuronal latency. Nat Commun.

[5] Zerboni L, Sobel RA, Lai M, Triglia R, Steain M, Abendroth A, Arvin A (2012). Apparent expression of varicella-zoster virus proteins in latency resulting from reactivity of murine and rabbit antibodies with human blood group a determinants in sensory neurons. J Virol.

[6] Laing KJ, Ouwendijk WJD, Koelle DM, Verjans GMGM (2018). Immunobiology of varicella-zoster virus infection. J Infect Dis.

[7] Thomsen MM, Tyrberg T, Skaalum K (2021). Genetic variants and immune responses in a cohort of patients with varicella zoster virus encephalitis. J Infect Dis.

[8] Herlin LK, Hansen KS, Bodilsen J (2021). Varicella zoster virus encephalitis in Denmark from 2015 to 2019-a Nationwide Prospective Cohort Study. Clin Infect Dis.

[9] Ochs HD, Edvard Smith CI (1996). X-linked agammaglobulinemia a clinical and molecular analysis. Rev Mol Med.

[10] St. Leger AJ, Koelle DM, Kinchington PR, Verjans GMGM (2021). Local Immune control of latent herpes simplex virus type 1 in ganglia of mice and man. Front Immunol.

[11] Etzioni A, Eidenschenk C, Katz R, Beck R, Casanova JL, Pollack S (2005). Fatal varicella associated with selective natural killer cell deficiency. J Pediatr.

[12] Mackay LK, Stock AT, Ma JZ, Jones CM, Kent SJ, Mueller SN, Heath WR, Carbone FR, Gebhardt T (2012). Long-lived epithelial immunity by tissue-resident memory T (TRM) cells in the absence of persisting local antigen presentation. PNAS.

[13] Khanna KM, Bonneau RH, Kinchington PR, Hendricks RL (2003). Herpes simplex virus-specific memory CD8+ T cells are selectively activated and retained in latently infected sensory ganglia. Immunity.

[14] Unger PPA, Oja AE, Khemai-Mehraban T, Ouwendijk WJD, Hombrink P, Verjans GMGM (2022). T-cells in human trigeminal ganglia express canonical tissue-resident memory T-cell markers. J Neuroinflammation.

[15] Vukmanovic-Stejic M, Sandhu D, Seidel JA (2015). The characterization of varicella zoster virus-specific T cells in skin and blood during aging. J Investig Dermatol.

[16] Laing KJ, Ouwendijk WJD, Campbell VL (2022). Selective retention of virus-specific tissue-resident T cells in healed skin after recovery from herpes zoster. Nat Commun.

[17] Verjans GMGM, Hintzen RQ, van Dun JM, Poot A, Milikan JC, Laman JD, Langerak AW, Kinchington PR, Osterhaus ADME (2007). Selective retention of herpes simplex virus-specific T cells in latently infected human trigeminal ganglia. PNAS.

[18] Ouwendijk WJD, Flowerdew SE, Wick D, Horn AKE, Sinicina I, Strupp M, Osterhaus ADME, Verjans GMGM, Hüfner K (2012). Immunohistochemical detection of intra-neuronal VZV proteins in snap-frozen human ganglia is confounded by antibodies directed against blood group A1-associated antigens. J Neurovirol.

[19] van Velzen M, Jing L, Osterhaus ADME, Sette A, Koelle DM, Verjans GMGM (2013). Local CD4 and CD8 T-cell reactivity to HSV-1 antigens documents broad viral protein expression and immune competence in latently infected human trigeminal ganglia. PLoS Pathog.

[20] Ouwendijk WJD, Geluk A, Smits SL, Getu S, Osterhaus ADME, Verjans GMGM (2014). Functional characterization of ocular-derived human alphaherpesvirus cross-reactive CD4 T cells. J Immunol.

[21] Laing KJ, Russell RM, Dong L, Schmid DS, Stern M, Magaret A, Haas JG, Johnston C, Wald A, Koelle DM (2015). Zoster vaccination increases the breadth of CD4+ T cells responsive to varicella zoster virus. J Infect Dis.

[22] Chronister W, Sette A, Peters B, Huang H, Davis MM (2022). Epitope-specific T cell receptor data and tools in the immune epitope database. T-Cell repertoire characterization. Methods in molecular biology.

[23] Braspenning SE, Sadaoka T, Breuer J, Verjans GMGM, Ouwendijk WJD, Depledge DP (2020). Decoding the architecture of the varicella-zoster virus transcriptome. MBio.

[24] Ouwendijk WJD, van den Ham HJ, Delany MW (2020). Alveolar barrier disruption in varicella pneumonia is associated with neutrophil extracellular trap formation. JCI Insight.

[25] Jing L, Laing KJ, Dong L (2016). Extensive CD4 and CD8 T cell cross-reactivity between alphaherpesviruses. J Immunol.

[26] Jing L, Haas J, Chong TM (2012). Cross-presentation and genome-wide screening reveal candidate T cells antigens for a herpes simplex virus type 1 vaccine. J Clin Investig.

[27] Koelle DM, Dong L, Jing L (2022). HSV-2-specific human female reproductive tract tissue resident memory T cells recognize diverse HSV antigens. Front Immunol.

[28] Ouwendijk WJD, Laing KJ, Verjans GMGM, Koelle DM (2013). T-cell immunity to human alphaherpesviruses. Curr Opin Virol.

[29] Balachandran N, Oba DE, Hutt-Fletcher LM (1987). Antigenic cross-reactions among herpes simplex virus types 1 and 2, Epstein-Barr virus, and cytomegalovirus. J Virol.

[30] Bradley P, Thomas PG (2019). Using T cell receptor repertoires to understand the principles of adaptive immune recognition. Annu Rev Immunol.

[31] Sidney J, Peters B, Sette A (2020). Epitope prediction and identification- adaptive T cell responses in humans. Semin Immunol.

[32] Laing KJ, Dong L, Sidney J, Sette A, Koelle DM (2012). Immunology in the clinic review series; focus on host responses: T cell responses to herpes simplex viruses. Clin Exp Immunol.

[33] Theil D, Derfuss T, Paripovic I, Herberger S, Meinl E, Schueler O, Strupp M, Arbusow V, Brandt T (2003). Latent herpesvirus infection in human trigeminal ganglia causes chronic immune response. Am J Pathol.

[34] Hüfner K, Derfuss T, Herberger S, Sunami K, Russell S, Sinicina I, Arbusow V, Strupp M, Brandt T, Theil D (2006). Latency of alpha-herpes viruses is accompanied by a chronic inflammation in human trigeminal ganglia but not in dorsal root ganglia. J Neuropathol Exp Neurol.

[35] van Velzen M, Ouwendijk WJD, Selke S, Pas SD, van Loenen FB, Osterhaus ADME, Wald A, Verjans GMGM (2013). Longitudinal study on oral shedding of herpes simplex virus 1 and varicella-zoster virus in individuals infected with HIV. J Med Virol.

[36] Johnston C, Magaret A, Son H (2022). Viral shedding 1 year following first-episode genital HSV-1 infection. JAMA.

[37] van Velzen M, Laman JD, KleinJan A, Poot A, Osterhaus ADME, Verjans GMGM (2009). Neuron-interacting satellite glial cells in human trigeminal ganglia have an APC phenotype. J Immunol.

[38] Levin MJ (2012). Immune senescence and vaccines to prevent herpes zoster in older persons. Curr Opin Immunol.

[39] Asada H (2019). VZV-specific cell-mediated immunity, but not humoral immunity, correlates inversely with the incidence of herpes zoster and the severity of skin symptoms and zoster-associated pain: The SHEZ study. Vaccine.

[40] Miller AE (1980). Selective decline in cellular immune response to varicella-zoster in the elderly. Neurology.

[41] Levin MJ, Smith JG, Kaufhold RM (2003). Decline in varicella-zoster virus (VZV)-specific cell-mediated immunity with increasing age and boosting with a high-dose VZV vaccine. J Infect Dis.

[42] Hope-Simpson RE (1965). The nature of herpes zoster: a long-term study and a new hypothesis. Proc R Soc Med.

