# Supplementary figures and images for: Varicella-zoster virus proteome-wide T-cell screening demonstrates low prevalence of virus-specific CD8 T-cells in latently infected human trigeminal ganglia
Source: J Neuroinflammation. 2023 Jun 12;20:141. doi: 10.1186/s12974-023-02820-y (PMC10259006; doi:10.1186/s12974-023-02820-y)

*POLR2A*

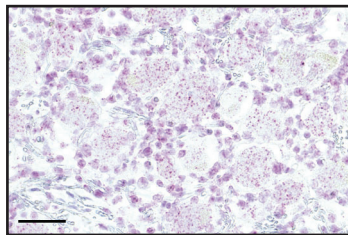

*DAPB*

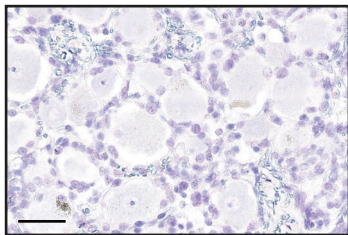

Supplement: Supplementary file 1 — Additional file 1: Figure S1. Control stainings for the in situ hybridization assays. Staining for the human gene POLR2A and the bacterial gene dapB were performed as positive and negative controls, respectively, for the in situ hybridization assays shown in main, Fig. 5; scale bar: 50 µm. [file 12974_2023_2820_MOESM1_ESM.pdf]

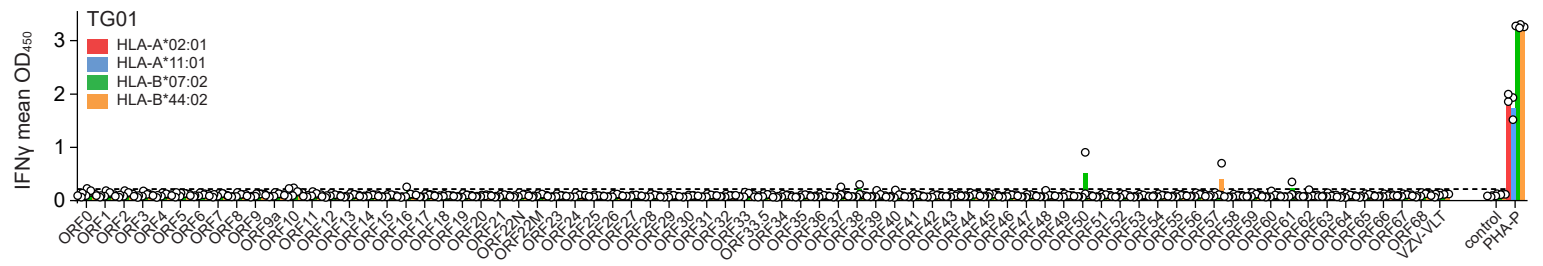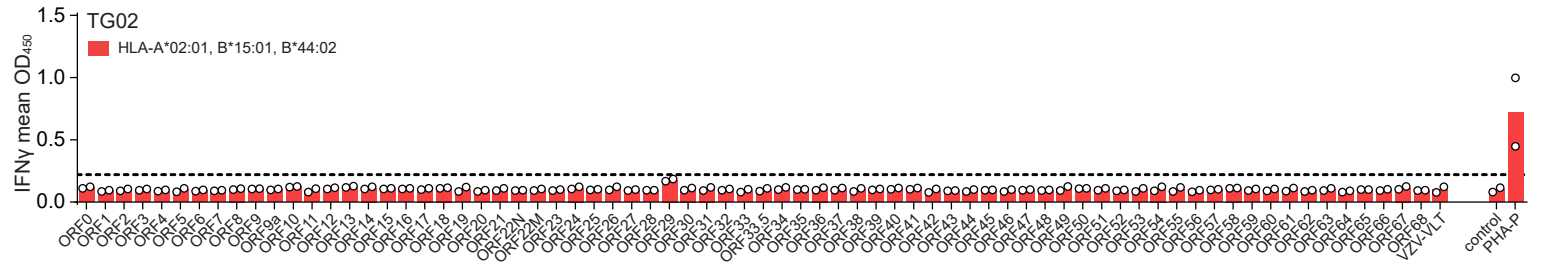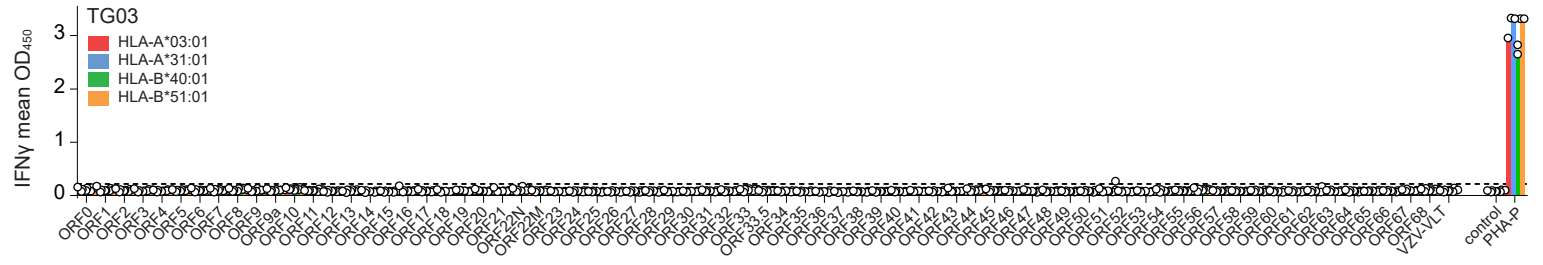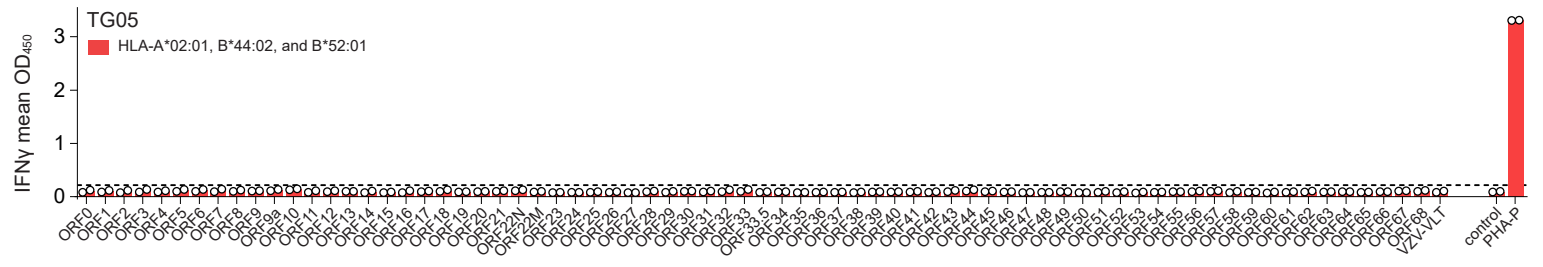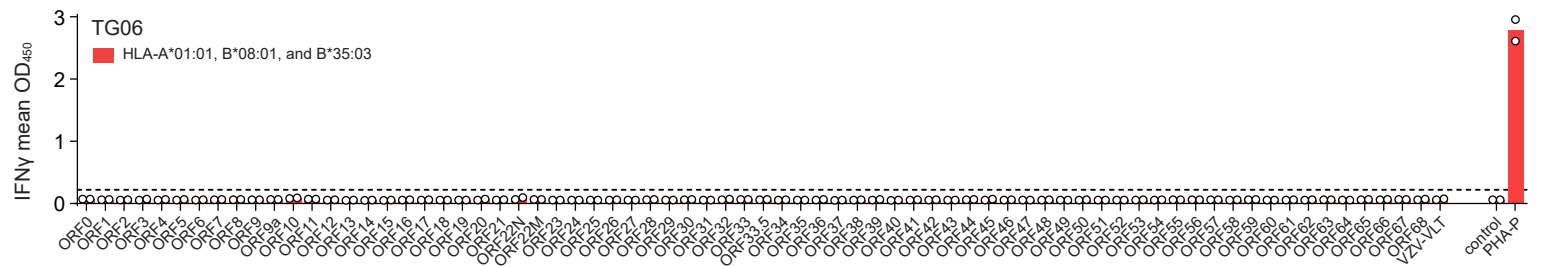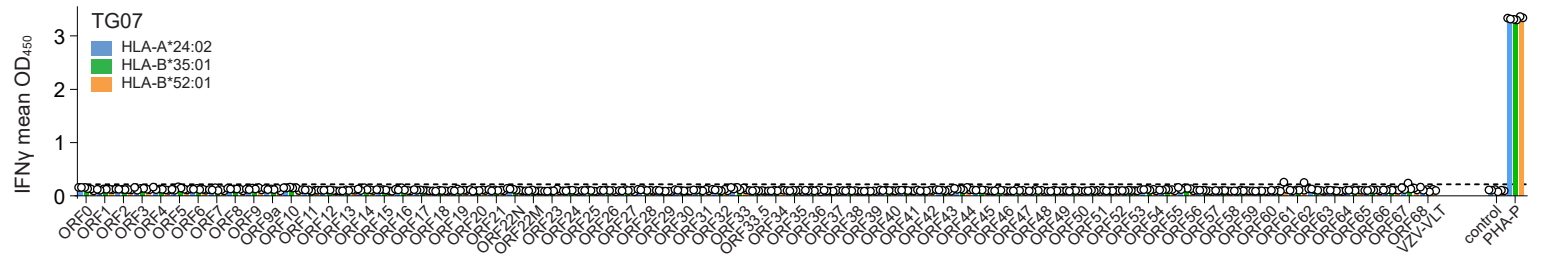

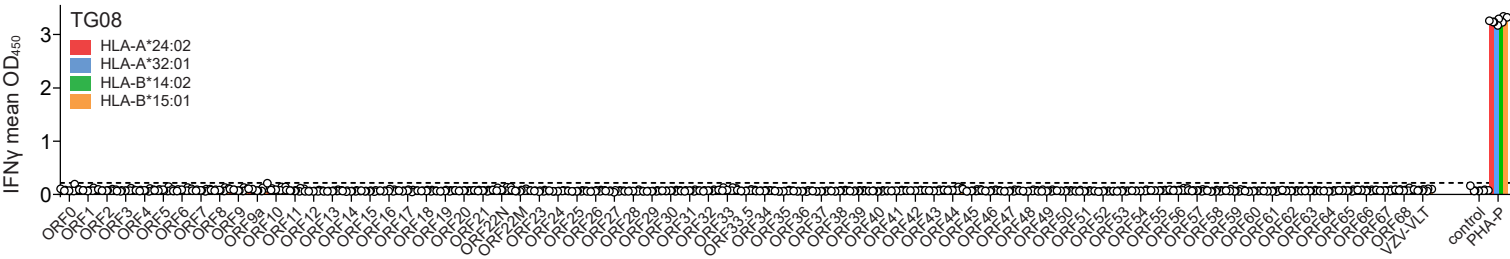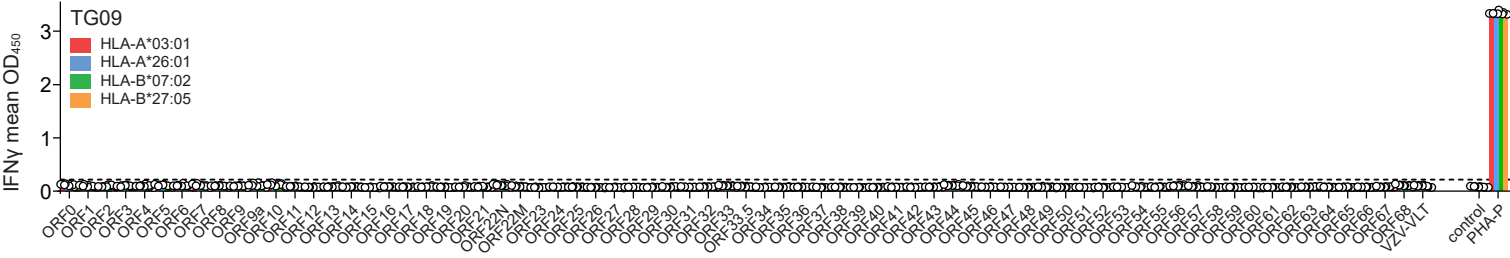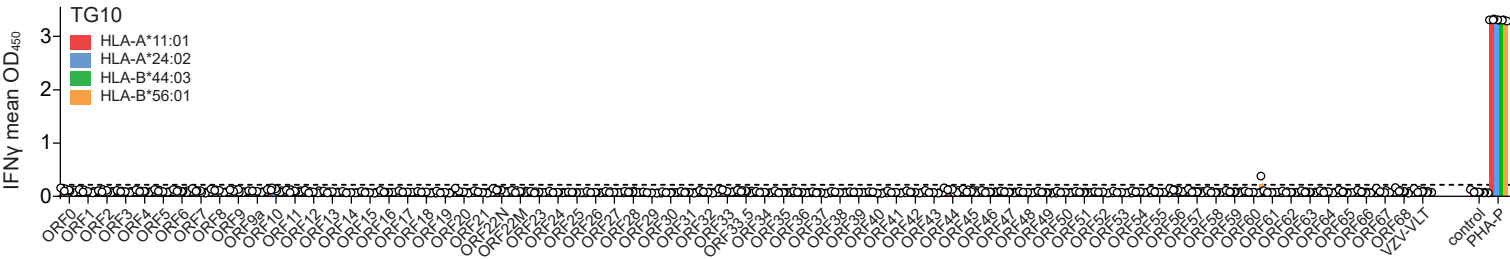

Supplement: Supplementary file 2 — Additional file 2: Figure S2. VZV antigen-specific responses of additional human TG-derived CD8+ T-cells. TG-derived T-cell lines of donor TG01–TG03 and TG05–TG10 were incubated with Cos-7 cells transfected with vectors encoding the indicated subject-specific HLA class I allele together with the individual VZV open reading frames. Empty vector and phytohemagglutinin were used as negative and positive controls, respectively. Levels of secreted IFNγ were determined by ELISA. Data are presented as the individual and mean OD450 values of two independent replicates. Horizontal dashed lines indicate the threshold of T-cell response set at twice the value of the empty vector control. Viral ORF nomenclature according to VZV reference strain Dumas. [file 12974_2023_2820_MOESM2_ESM.pdf]
