# Supplementary material for: Varicella-zoster virus proteome-wide T-cell screening demonstrates low prevalence of virus-specific CD8 T-cells in latently infected human trigeminal ganglia
Source: J Neuroinflammation. 2023 Jun 12;20:141. doi: 10.1186/s12974-023-02820-y (PMC10259006; doi:10.1186/s12974-023-02820-y)
Supplement: Supplementary file 3 — Additional file 3: Table S1. General characteristics and HLA genotype of study subjects used for T-cell analysis. [file 12974_2023_2820_MOESM3_ESM.pdf]

Table S1. General characteristics and HLA genotype of study subjects used for T-cell analysis.

ID	Age (yrs)	Sex	Cause of death	Neurological disease	PMI	Infection status VZV	HSV-1	HLA-A	HLA-B	HLA-C	HLA-DRB1	HLA-DQB1
TG01	76	Female	Palliative sedation	Alzheimer's disease	6:20	Pos	Pos	02:01<br>11:01	07:02<br>44:02	05:01<br>07:02	04:01<br>15:01	03:01<br>06:02
TG02	77	Female	Renal insufficiency	Pick's disease	4:30	Pos	Pos	02:01<br>02:01	15:01<br>44:02	03:01<br>03:01	04:01<br>15:01	ND
TG03	80	Female	Dehydration	Progressive supranuclear palsy	6:45	Pos	Pos	03:01<br>31:01	40:01<br>51:01	ND	ND	ND
TG04	71	Male	Airway infection	Non-demented control	5:45	Pos	Pos	01:01<br>02:01	07:02<br>08:01	ND	ND	ND
TG05	90	Female	Dehydration	Alzheimer's disease	4:30	Pos	Pos	02:01<br>02:01	44:02<br>52:01	ND	ND	ND
TG06	82	Female	Pneumonia	Dementia	4:00	Pos	Pos	01:01<br>01:01	08:01<br>35:03	04:01<br>07:01	01:01<br>03:01	02:01<br>05:01
TG07	56	Female	Euthanasia	Multiple sclerosis	4:45	Pos	Neg	11:01<br>24:02	35:01<br>52:01	03:03<br>12:02	01:01<br>13:01	05:01<br>06:03
TG08	84	Male	Pneumonia	Parkinson's disease	9:00	Pos	Pos	24:02<br>32:01	14:02<br>15:01	05:01<br>07:02	01:01<br>01:02	05:01<br>05:01
TG09	81	Female	Sepsis	Multiple sclerosis	7:17	Pos	Pos	03:01<br>26:01	07:02<br>27:05	02:02<br>07:02	04:04<br>15:01	03:02<br>06:02
TG10	99	Female	Pneumonia	Alzheimer's disease	4:15	Pos	Pos	11:01<br>24:02	44:03<br>56:01	01:02<br>16:01	03:01<br>07:01	02:01<br>02:02

TG, trigeminal ganglion; HLA, human leukocyte antigen; PMI, post-mortem interval (hours:minutes); ND, not determined; Pos, positive; Neg, negative. Infection status was determined by plasma IgG ELISA and/or qPCR on TG-derived DNA, indicated as positive if either or both of the two tests yielded a positive result.
