# Supplementary material for: Varicella-zoster virus proteome-wide T-cell screening demonstrates low prevalence of virus-specific CD8 T-cells in latently infected human trigeminal ganglia
Source: J Neuroinflammation. 2023 Jun 12;20:141. doi: 10.1186/s12974-023-02820-y (PMC10259006; doi:10.1186/s12974-023-02820-y)
Supplement: Supplementary file 4 — Additional file 4: Table S2. General characteristics of the study subjects used for histological analysis in main, Fig. 5. [file 12974_2023_2820_MOESM4_ESM.pdf]

Table S2. General characteristics of the study subjects used for histological analysis in main Figure 5.

ID	Age (yrs)	Sex	Cause of death	Neurological disease	PMI	Infection status	
						VZV	HSV-1
TG11	63	Female	Gastrointestinal bleeding	Dementia	04:00	Pos	Neg
TG12	62	Female	Cachexia and pulmonary insufficiency	Multiple sclerosis	12:35	Pos	Neg
TG13	81	Female	Cachexia and dehydration	Alzheimer's disease	03:35	Pos	Neg
TG14	64	Female	Cachexia	Dementia	07:30	Pos	Neg
TG15	63	Female	Metastatic breast cancer	Schizophrenia	05:00	Pos	Pos

TG, trigeminal ganglion; PMI, post-mortem interval (hours:minutes); Pos, positive; Neg, negative. Infection status was determined by qPCR on trigeminal ganglion-derived DNA.
