# Supplementary material for: Varicella-zoster virus proteome-wide T-cell screening demonstrates low prevalence of virus-specific CD8 T-cells in latently infected human trigeminal ganglia
Source: J Neuroinflammation. 2023 Jun 12;20:141. doi: 10.1186/s12974-023-02820-y (PMC10259006; doi:10.1186/s12974-023-02820-y)
Supplement: Supplementary file 5 — Additional file 5: Table S3. Primers used to generate the VZV ORF29 truncated fragments. [file 12974_2023_2820_MOESM5_ESM.pdf]

Table S3. Primers used to generate the VZV ORF29 truncated fragments.

**Fragment**	**Forward Primer**	**Reverse Primer**	**Base pair range**	**Amino acid range**	**Size (AA)**
ORF29-A	ggggacaagtttgtacaaaaaagcaggcttcATGGAAAATACTCAGAAGACTGT	ggggaccactttgtacaagaaagctgggtcTTAAACATCGGGCATGAAAAGTTGT	1-610	1-203	203
ORF29-B	ggggacaagtttgtacaaaaaagcaggcttcTTTCTGCATTATGGAGGACTC	ggggaccactttgtacaagaaagctgggtcTTAAGCGGTATATGACCCCAGAGCG	514-1110	172-370	199
ORF29-C	ggggacaagtttgtacaaaaaagcaggcttcGCAGAAGTTATATTCAACACTGG	ggggaccactttgtacaagaaagctgggtcTTATAAATATGGAGCATAGTTTCCT	988-1663	330-554	225
ORF29-D	ggggacaagtttgtacaaaaaagcaggcttcCTTAGACAACGCATGCCGCG	ggggaccactttgtacaagaaagctgggtcTTAGTTACGAAAGTTCCGCCCCTCA	1546-2114	516-704	189
ORF29-E	ggggacaagtttgtacaaaaaagcaggcttcACCAATTTTTTAGTTAAACGAACAC	ggggaccactttgtacaagaaagctgggtcTTAAACAGCTGCAATAGTGGTA	2002-2600	668-866	199
ORF29-F	ggggacaagtttgtacaaaaaagcaggcttcAAATCCCCCAACCCGCAGTG	ggggaccactttgtacaagaaagctgggtcTTAACCTCCTCTAGGACATGCTATTAT	2500-3099	834-1033	200
ORF29-G	ggggacaagtttgtacaaaaaagcaggcttcGCAGGGAATTGGAGCGGTT	ggggaccactttgtacaagaaagctgggtcTTAAATCATTTCCATTGTAAT	3001-3615	1001-1204	199

Note: Gateway attB primers in lowercase font
