# Supplementary material for: Varicella-zoster virus proteome-wide T-cell screening demonstrates low prevalence of virus-specific CD8 T-cells in latently infected human trigeminal ganglia
Source: J Neuroinflammation. 2023 Jun 12;20:141. doi: 10.1186/s12974-023-02820-y (PMC10259006; doi:10.1186/s12974-023-02820-y)
Supplement: Supplementary file 6 — Additional file 6: Table S4. Homology of HSV-1 CD8 T-cell epitope-containing regions with orthologous VZV proteins. [file 12974_2023_2820_MOESM6_ESM.docx]

**Table S4.** Homology of HSV-1 CD8 T-cell epitope-containing regions with orthologous VZV proteins.

| **Epitope ID** | **Virus / Gene** | **HLA restricting allele** | **Protein sequence**  **(amino acid boundary numbers)*** | **Binding rank for minimal epitope or homolog** |
| --- | --- | --- | --- | --- |
| 1 | HSV-1 / RL2 | A*02:01 | 0637 – GGLTR**YLPISGVSSV**VALSP - 0656 | 0.11 |
|  | VZV / ORF61 |  | No homology found in ORF61 | Not applicable |
| 2 | HSV-1 / ICP4 | A*03:01 | 1091 – EAAHA**RLYPDAPPLR**LCRGG – 1110 | 0.02 |
|  | VZV / ORF62 |  | 1013 – ETAFAN**LYP**GEQ**PL**CLCRGG – 1032 | 15 |
| 3 | HSV-1 / UL46 | B*07:02 | 0381 - LFPTM**TAPSWARMEL**SIKAW - 0400 | 0.07 |
|  | VZV / ORF12 |  | 0396 - LVRTMSTH**SW**VVL**E**TSTHMW - 0415 | 33 |
| 4 | HSV-1 / UL29 | A*02:01 | 1091 – SLTDD**EFLARDLEEL**HDQII - 1110 | 2.3 |
|  | VZV / ORF29 |  | 1090 – ALLED**E**Y**L**SEEMM**EL**TARAL - 1111 | 0.98 |
| 5 | HSV-1 / UL1 | A*01:01 | 0063 - INYA-**LIDGIFLRY**HCPGLD - 0081 | 0.01 |
|  | VZV / ORF60 |  | 0064 – SNLSEALS**GI**VVKTKCPVPE – 0082 | 16 |
| 6 | HSV-1 / UL53 | A*01:01 | 0196 – LVQLF**ETDPVTFLY**HRPAIG - 0215 | 0.01 |
|  | VZV / ORF5 |  | 0194 – MSKVFNA**DP**IS**FLY**MHKGVT - 0213 | 0.01 |
| 7 | HSV-1 / UL47 | A*29:02 | 0503 - YALHT**ALATVTLKY**ARACGD - 0522 | 0.01 |
|  | VZV / ORF11 |  | 0645 – EAVRT**A**ATNFMIEFADCYKE - 0564 | 1.8 |
| 8 | HSV-1 / UL48 | A*01:01 | 0085 - NEDLF**SALPTNADLY**RECKF - 0104 | 0.28 |
|  | VZV / ORF10 |  | 0089 - NEDLF**S**CF**P**I**N**E**DLY**SDMMV - 0108 | 0.58 |
| 9 | HSV-1 / UL48 | B*40:01 | 0158 – HAELR**AREESYRTVL**ANFCS - 0178 | 0.2 |
|  | VZV / ORF10 |  | 0162 – TVELR**AREE**A**Y**TKL**L**VTYCK – 0181 | 0.49 |
| 10 | HSV-1 / UL48 | A*01:01 | 0471 – ADFEFEQMF**TDALGIDEY**GG – 0490 | 0.5 |
|  | VZV / ORF10 |  | No homology found in ORF10 | Not applicable |

HSV-1 protein and HLA class I allele restriction of these CD8 T-cell epitopes was previously identified in human TG-derived T-cell lines [19].

* The HSV-1 CD8 T-cell epitope sequence is underlined, bold, and red. Amino acid homologies with the orthologue VZV protein sequence are shown in red. The sequences are derived from HSV-1 strain 17 (Genbank Accession number NC_001806.2) or VZV strain Dumas (Genbank Accession number NC_001348.1); HLA, human leukocyte antigen.
